# Evaluating the effectiveness of anti-Nogo treatment in spinal cord injuries

**DOI:** 10.1186/s13064-020-0138-9

**Published:** 2020-01-09

**Authors:** Raihan Mohammed, Kaesi Opara, Rahul Lall, Utkarsh Ojha, Jinpo Xiang

**Affiliations:** 1 Department of Medicine, School of Clinical Medicine, University of Cambridge, Hills Rd, Cambridge, CB2 0SP UK; 2 Faculty of Medicine, Imperial College London, London, UK

**Keywords:** Axon, CNS, Regeneration, Myelin, Nogo

## Abstract

As humans, we cannot regenerate axons within the central nervous system (CNS), therefore, making any damage to it permanent. This leads to the loss of sensory and motor function below the site of injury and can be crippling to a person’s health. Spontaneous recovery can occur from plastic changes, but it is minimal. The absence of regeneration is due to the inhibitory environment of the CNS as well as the inherent inability of CNS axons to form growth cones. Amongst many factors, one of the major inhibitory signals of the CNS environment is the myelin-associated Nogo pathway. Nogo-A, Nogo-B and Nogo-C (Nogo), stimulate the Nogo receptor, inhibiting neurite outgrowth by causing growth cones to collapse through activation of Rho Kinase (ROCK). Antibodies can be used to target this signalling pathway by binding to Nogo and thus promote the outgrowth of neuronal axons in the CNS. This use of anti-Nogo antibodies has been shown to upregulate CNS regeneration as well as drastically improve sensory and motor function in both rats and primates when coupled with adequate training. Here, we evaluate whether the experimental success of anti-Nogo at improving CNS regeneration can be carried over into the clinical setting to treat spinal cord injuries (SCI) and their symptoms successfully. Furthermore, we also discuss potential methods to improve the current treatment and any developmental obstacles.

## Inhibitors of spinal regeneration

It is unclear whether CNS regeneration was an ancestral trait lost during evolution or an adaptive trait developed over time. The fact that it is present in fish and some amphibians would suggest that the trait has diminished or was lost over time [1]. In the context of survival, CNS regeneration does not provide a major advantage. If an animal is unable to move until the spinal cord regenerates, then it is vulnerable to attack by predators and unable to seek resources. Therefore, it would make sense to trade regenerative ability for the complexity of connections and more direct motor control from the cortex seen in higher species. This would allow us to build and develop alternate strategies for improving survival chances such as fortified shelters or tools. Humans undergo extensive neurogenesis during development, leading to the formation of the most complex and intricate circuits known to man. This fetal plasticity of the CNS is maintained into neonatal life with extensive recovery observed following an injury [2]. Despite this, as adults, we can only regenerate peripheral nerves to a certain amount and do not have the ability to reform CNS connections following a spinal cord injury [3]. Corticospinal axons will only extend for 1 millimetre caudal to the injury and so cannot contribute to the recovery process. The only functional recovery following SCI comes from sprouting and reorganisation of axons rostrally and caudally, as well development of compensatory movement patterns [4]. This recovery, however, is often minimal and heavily depends on the extent of the lesion. Prognosis is best when there is more spared tissue through which bypass circuits can form.

With no more real danger of predators and developed healthcare systems, it would be highly desirable to regain the ability to recover from spinal cord injuries. This is because such injuries can be extremely debilitating in everyday life. Damage to axons in the spinal cord means loss of sensation as well as motor and autonomic outputs caudal to the damage. In paraplegic patients, this means loss of bowel and urinary continence as well as the lack of sexual function. Quadriplegic patients can also experience cardiovascular problems caused by the loss of the thoracic sympathetic chain [5]. In early days of treating SCI, most patients died from developing urinary tract infections or pressure sores [6]. In today’s world, however, most sufferers of spinal cord injury survive, and the initial damage is minimised. Treatment now concentrates mostly on controlling bladder dysfunction and neuropathic pain [7]. This is most often coupled with locomotor training such as walking on a treadmill [8]. Together modern treatments aim to improve the patient’s quality of life as much as possible, but with the lack of regeneration in the CNS, there is only so much that can be done.

There are a number of reasons for this regenerative disparity between peripheral and central nerves. Initially, it was believed that CNS axons have an intrinsic inability to regenerate. However, this has since been disputed multiple times. Experiments have shown that when the spinal cord is cut the nerves can regenerate into a peripheral graft demonstrating that CNS axons have at least some ability to regenerate [9]. The same peripheral nerve, however, will not regenerate into the CNS, suggesting that the limiting factor is the environment of the CNS. In the PNS the cut axon degenerates distally to the cut in an active process known as Wallerian degeneration, leaving the sheath and surrounding Schwann cells to support the growth of the new axon. The proximal end of the cut then forms a growth cone as the end is sealed and which can extend using actin filaments [10]. In contrast, in the CNS, the growth cone is prevented from forming and remaking connections. Firstly, as CNS neurones mature they stop producing necessary adhesion molecules. In addition, selective transport of molecules into CNS axons means many growth factors are excluded from the axon reducing the axons overall regenerative ability [11]. Secondly, a glial scar forms at the site of injury. This is a protective barrier which reduces inflammation and allows the blood brain barrier to reform. However, it is also physically stops axons from crossing the site of lesion [12]. The extracellular matrix of central neurones itself is also quite non-permissive with many chondroitin sulphate proteoglycans (CSPGs) forming an impassable mesh [13].

Finally, there are a number of inhibitory signalling pathways which also cause the collapse of the growth cone. The idea that the CNS environment was inhibitory was cemented by Schwab. He showed that neurones would regenerate into sciatic nerve explants, but not optic nerve ones, regardless of supplying them with growth factors [14]. Three years later Caroni and Schwab found that myelin-associated inhibitors NI-250 and NI-35 were causative of the non- permissive nature of the CNS [15]. These molecules were later purified, and DNA sequenced by another group and subsequently came to be known as Nogo [16].

The gene encoding Nogo has three protein products: Nogo-A, Nogo-B and Nogo-C. Of these three Nogo-A was the most potent inhibitor in the CNS with the other two transcripts of the gene having some peripheral distribution. Nogo-A has an activating extracellular domain named Nogo-66 as well as a smaller activating region in the extracellular N-terminal known as amino-Nogo. Nogo-66 exerts its main function at the NgR1 receptor while S1PR2 has recently been identified as the receptor for amino-Nogo [17]. Though Nogo-A is the strongest activator of NgR1, it is one of three myelin-associated proteins along with MAG and OMgp originally thought to stimulate the receptor [18]. Furthermore, it has now been shown that CSPGs of the extracellular matrix also stimulate NgR1 [19]. The binding of all these molecules to NgR activates a signalling cascade via the Rho family GTPases which eventually leads to growth cone collapse [20]. It is now believed that this pathway primarily stabilises connections and limits turnover in the brain in the transition to adulthood. The decrease in plasticity and dendritic turnover usually seen in adolescent rats coincides with the appearance of NgR1 ligands. Furthermore, Nogo knockout mice maintained this plasticity into adulthood and deletion of NgR1 in adult mice restored it [21]. Therefore, it could be said that the Nogo pathway limits rearrangements regardless of whether they are experience or injury driven [22].

## Effects of anti-Nogo therapy

One possible strategy for increasing a central neurones ability to regenerate would be to block this signalling pathway thus preventing growth cone collapse. One way of achieving this would be to use antibodies. Since the development of monoclonal antibodies, they have been redesigned to serve as experimental tools as well as targeting a wide variety of signalling pathways. After Caroni and Schwab first identified the NI-35 and NI-250 molecules they raised antibodies against these to see if this would promote axonal regeneration [15]. In their experiment, they examined neurite growth in vitro. As optic nerve explants do not usually support the growth of neurites in vitro, they first identified parts of the optic nerve where laminin was present. They knew laminin to support neurite growth in vitro from experiments using the sciatic nerve. Despite the presence of laminin around blood vessels and on the pial membrane no neurite growth was found after 5 weeks. They went on to treat these explants with the IN-1 antibody and compared these to controls where a non-reactive antibody O_1_ was used. They found that IN-1 significantly promoted the growth of sensory and sympathetic neurites into the optic nerve explant. Their results showed that neurite growth of 3 mm or more was witnessed five out of six times in explants treated with IN-1 compared with one out of six in control cultures.

However, this study was very limited in its usefulness. With a small number of nerve explants in vitro, it was impossible to say at this point whether this would have any potential in treatment. However, 2 years later the experiment was performed in vivo. This was achieved by first implanting young rats with tumours producing IN-1 and then transecting the cortico-spinal tract [23]. With all previous studies showing a complete lack of regeneration, it was a breakthrough to see IN-1 treated rats with fine axons extending up seven to 11 mm caudal to the lesion. Massive sprouting was also observed at the site of the lesion. This contrasts with untreated rats where similar sprouting occurred though barely ever exceeded 1 mm. Though this study confirmed the effects of the antibody on neural regeneration in vivo, it still did not tell us whether it could serve as a potential treatment.

It was not long before another team used IN-1 to investigate the longevity of these anatomical connections as well as their potential in functional recovery [24]. Young rats were given a mid-thoracic spinal lesion and similarly to the previous study mentioned, they were implanted with an IN-1 secreting tumour. Rats were then given 4 to 6 weeks before behavioural training. As expecting neuronal projections increased caudally and were also shown to increase in the serotonergic and adrenergic systems. These projections persisted for the whole length of testing surviving 12 weeks. The rats were later tested for functional recovery against control healthy rats, lesions only rats, and lesioned rats which received control antibody.

Contact placing, where neurons extend their axons to make contact with their target, was completely absent in the untreated rats as well as those treated with control antibody. However, in rats treated with IN-1, 80% elicited low threshold contact placing responses. Stride length was also significantly affected without IN-1 treatment in the limb ipsilateral to the lesion. With treatment though it was not significantly different to control healthy rats. Contact placing was later lost following cortical ablation suggesting that the functional recovery was indeed caused by the regeneration of corticospinal pathways. On the other hand, the cortical ablation was found not to affect the stride length significantly. This would suggest that pathways controlling stride length do not originate in the cortex. Instead, this marked improvement in IN-1 treated animals could be due to rewiring of connections at the level of the brain stem. Unfortunately, in other aspects of motor function, no recovery was observed. For example, for more precise tasks like foot placement on grid runways the error rate for IN-1 treated rats and HRP treated rats was the same.

Since then many studies have expanded on this each time reinforcing the conclusion that anti-Nogo antibodies improve functional recovery. Most importantly, similar results were reproduced in primates. Monkeys were required to grab food pellets on a board which required the opposition of index finger and thumb in both horizontal and vertical orientations [25]. This was designed to assess their fine finger movements, and dexterity and so is a particularly useful indication of the clinical usefulness of anti-Nogo treatment. Both horizontal and vertical placing times were significantly reduced after treatment thus showing that the treatment is equally effective in higher mammals.

Though the focus of most studies was assessing functional recovery of motor and sensory input, treatment has been found to improve other effects of SCI as well. For example, treatment with anti- Nogo antibodies also reduced muscular spasms usually experienced by people living with SCI. These most likely arise as a result of the incorrect wiring of spinal circuitry following injury. Rats treated with anti-Nogo antibodies had a much lower prevalence of spasms, and the spasms were less likely to be triggered when the rats were exposed to cold water. Though unfortunately, the severity of the spasms was not significantly different between the groups [26]. There has not been much work concerning the effect of Nogo in the autonomic system. However, some studies do suggest an involvement of Nogo in the parasympathetic system. Mice treated with antibodies have been reported to have a better insulin secretion associated with a higher parasympathetic input and a greater sensitivity to carbachol [27]. This could mean that following SCI treatment might improve certain autonomic functions. In one experiment it was noted that control of bladder function returned to rats treated with anti-Nogo on average a week earlier than control groups [28]. Finally, none of these studies reported any side effects such as hyperalgesia or allodynia which can often present when incorrect connections are made in the nervous system.

Thus, results of studies using anti-Nogo have been very promising. Recovery of motor function has been consistently demonstrated in rats as well as primates with fine finger control returning after treatment. Furthermore, no sensory side effects such as hyperalgesia or allodynia were observed. The treatment also alleviated other symptoms of SCI such as bladder dysfunction and muscle spasms making it ideal for application in a clinical setting.

## Mechanism of action

Re-lesioning of the spine in Bregman et al. highlighted that the recovery might not be due to axons growing through the site of the lesion. Instead, might it be the increase in compensatory pathways that are responsible for the increase in functional recovery? Early findings have revealed that IN-1 indeed increased plasticity in supraspinal pathways. Following a lesion of the corticospinal tract, both the lesioned and unlesioned fibres showed appropriate sprouting after applying IN-1. One team found that after transecting the spinal cord, some of the transected hindlimb CST axons entered cervical grey matter where they made connections to short and long propriospinal axons [29]. They observed that contacts with long propriospinal axons that crossed the lesion were maintained over the course of the study. Retrograde tracing then confirmed that these long propriospinal axons made connections caudally with the hindlimb motorneurons. This created a new circuit within the spinal cord which enabled the rats to regain voluntary control of the hindlimb. The short propriospinal axons were not maintained, however, suggesting selection and maintenance of significant connection. Pharmacological destruction of these spinal interneurons was found to abolish the recovery of hindlimb function [30]. Another team found that rats treated with IN-1 had significantly more fibres crossing the midline at the level of the red nucleus and pons [31]. These fibres were shown to originate in the area of the cortex associated with forelimb movements. The hypothesis that this crossing over was responsible for recovery was reinforced by a repeated lesion to the tract. This should disrupt any regenerating fibres, and it was found not to have any effect on the functional recovery.

Another study also investigated this possible involvement of the rubrospinal tract in recovery [32]. Following a lesion and treatment with IN-1, stimulation of the cortex evoked contraction in the forelimb muscle. The increased latency in conduction following a lesion was suggestive of an increase in the number of synapses between the cortex and muscle. To test whether this new pathway involved the rubrospinal tract, both the red nuclei were injected with muscimol. This abolished the forelimb response to electrical stimulation of the cortex seemingly confirming the involvement of the rubrospinal tract in reconnecting the cortex to the periphery. They also found that excitability in the red nucleus has been increased following the reorganisation of the pathway. All these findings parallel the pattern of recovery seen in neonatal rats. Following SCI they show an increase in sprouting and crossing over of fibres, thus supporting the hypothesis that Nogo drives the decrease in CNS plasticity during adolescence.

Next, looking to the motor cortex, it would seem anti-Nogo treatment does not positively affect reorganisation. A group of monkeys had the area of the motor cortex responsible for digit movements mapped [33]. All of the monkeys had this area of the motor cortex then lesioned with a group of them receiving anti-Nogo treatment as well. The treated group showed a considerable improvement in dexterity tests and recovery of hand function. However, when the cortex was later remapped, there was no significant difference between any of the monkeys. Forearm areas of the cortex were not partly reassigned to the digits, and the areas of the lesions that remained excitable were not related to the degree of functional recovery.

Thus, recovery of function facilitated by anti-Nogo antibodies is likely caused by rewiring of remaining motor circuits. With increased plasticity, more fibres can cross over in the brainstem as well as spinal cord and create new pathways. Certain movements are affected by repeated disruption of the CST pathway whereas others seem to be resistant. This suggests some motor patterns could be controlled by the rubrospinal pathway following injury with others relying on the CST forming intraspinal bypass routes using propriospinal neurones. Therefore, when treatment strategies should concentrate on maximising the anti-inhibitory effects in these supraspinal and intraspinal segments of the motor pathways.

Finally, it is important to point out that the first anti-Nogo antibody, IN-1, is very different from those used in more recent studies. Neither of these antibodies have been carefully analyzed for their targets and so it is not fully known whether the regenerative capacity mentioned above is due to i) additional regenerative effects of the anti-Nogo antibodies (e.g. IN-1) beyond Nogo neutralization and/ or ii) additional roles of Nogo and the Nogo receptor beyond growth inhibition. For example, IN-1 has been shown to enhance the sprouting of spared neurons following a lesion and increases the plasticity of surrounding neurons to achieve compensatory sprouting [34]. Based on the largely intracellular (endoplasmic reticulum) localization of Nogo isoforms, they may be involved in the secretory pathway, such as functioning as chaperones for the trafficking and maturation of certain proteins. Evidence from He et al. has implicated Nogo-B in the regulation of B-secretase activity [35], and has been shown to modulate apoptosis, based on its interaction with pro-apoptotic proteins of the BCL-2 family [36]. As such, immunotherapy with anti-Nogo antibodies may have off-target effects and impact intrinsic neuronal regenerative capacity unrelated to their inhibitory signalling roles. These unintended effects may also be responsible for the side effects of IN-1 addition; hyperalgesia has been associated with the aberrant growth and sensitization of C, Aδ and Aβ nociceptive fibres, which may be from apoptotic reorganisation of neural circuits [37].

## Factors affecting outcome of therapy

However, increased plasticity and sprouting of axons itself is not particularly useful. The correct connections must be made and maintained for the new circuits to be functional. One study showed that when chondroitinase was used to increase plasticity in lesioned rats only the ability which was subsequently trained improved. In contrast, other abilities suffered as untreated rats performed better in tasks which were not included in the training regime of the treated groups [38]. The different pathways by which movements can be achieved will likely compete with each other during spinal and cortical reorganisation. This could lead to certain areas of the motor cortex and the patterns they contain to be extinguished. Once again, a comparison can be drawn to neonatal rats and babies. As mentioned before neonates exhibit remarkable recovery in motor functions following SCI. However, due to areas of cortex competing against each other, this can lead to permanent deficits in cognitive abilities.

Another factor to consider when trying to assess the effectiveness of these antibodies would be how the timing of the antibody treatment would affect its efficacy. One study aimed to answer this question by lesioning thoracic spinal cord in rats then observing the anatomical and functional recovery after delaying treatment [39]. As a comparison, some rats were still given anti-Nogo-A antibody acutely as well as control IgG. They noted that the potential for recovery was decreased as the delay before treatment increased. The axons regenerated over a distance of many millimetres in both the acute group and one-week delayed group. However, when treatment was delayed by 2 weeks, this was not observed. This was mirrored by the functional recovery. In swimming tests and crossing narrow beams, only the rats from the acute and one-week delayed group displayed significant recovery.

Another study reported that when using soluble NgR to target the binding domain of Nogo in chronic spinal cord contusion results were seen even with treatment beginning 4 to 6 months post injury [40]. The treatment did not promote longer sprouting of corticospinal fibres, which would be consistent with Gonzenbach et al.’s findings. However, some of the rats became weight bearing, and an increase of raphespinal axons was observed. This increase in serotonin fibre density correlated with the gradual recovery of locomotor functions. These results are reinforced by the functional recovery of rats which received anti-Nogo treatment 9 weeks after ischaemic stroke [41]. In this group increased crossing over at the red nucleus was observed. However, it should be said in the studies where treatment was delayed, the improvements were gradual and of a lesser extent than rats treated acutely. Nonetheless, more studies should investigate the optimal window of time for effective treatment.

As we saw from Garcia-Alias et al. dexterity training had a substantial impact on the recovery of different functions [38]. We should then consider how this training should be timed with respect to antibody treatment and how changing the periodization of training affects the functional recovery. One experiment subjected a group of rats to both training and antibody treatment concurrently, directly after they have been lesioned [42]. They were later compared to a group receiving the antibody treatment first then dexterity training after a two-week delay, as well as two control groups. The group receiving the antibody treatment parallel with dexterity scored the worst of all four groups. This group was observed to have an aberrant growth pattern which led to incorrect motor patterns forming during re-wiring of the spinal circuitry. The group which was treated first then trained later performed significantly better than the controls as expected.

Recently it was observed once again that treatment with anti-Nogo followed by training was the most efficient strategy for increasing recovery after an incomplete thoracic lesion [43]. Climbing errors and paw dragging in rats were both reduced which is important as the CST is crucial in digit movement confirming its involvement in the recovery. Although few fibres reached the lumbar cord, the group hypothesised that indirect connections caudal to the lesion were responsible for the restoration of precise paw control. This was reinforced by the observation of increasing primary afferents and their connections to motor neurones caudal to the lesion. Interestingly, sequential therapy resulted in lower levels of Ia motor neurone sprouting than the anti-Nogo therapy on its own similar to Bareyre et al.’s observation with propriospinal neurones [29]. This was first explained by Wahl et al.’s group who suggested that sequential therapy “may then help to shape the spared and new circuits by selection and stabilisation of functional connections and pruning of the non-functional ones [42]. Lastly, much like other findings so far, no allodynia or hyperalgesia were reported.

## Obstacles to development

As with any field, research should be reproducible to confirm the findings. Unfortunately, with much research regarding SCI, this is not the case. When a number of studies were repeated, most of them did not reproduce the original results [44]. There is also an ongoing debate about the genetic evidence for a role of Nogo in inhibiting axon regeneration and plasticity. Investigations have been performed on mice with targeted disruption of Nogo and the Nogo receptor, with results ranging from significant regenerative improvement (supporting antibody immunotherapy) to no improvement. For example, Zheng et al. did not observe any more significant regeneration in CST fibre sprouting in Nogo-A/B/C knockout mice compared with wild type mice [45]. However, other teams have shown either significant or moderate fibre regeneration in young Nogo A/B knockout mice [46, 47]. compare with heterozygous or wild type mice. More recent genetic disruption of the Nogo receptor have shown similarly disappointing results; Mice with Nogo receptor deficiency had improved raphespinal and rubrospinal axon regeneration [48], 2004 and no improvement in terms of corticospinal regeneration compared with wild type controls [49]. The varying degrees of regenerative capacity of these mice were attributed to differences in their genetics.

Furthermore, the effect of injury on the spinal cord and its connections is complex. Increasing plasticity by itself is useless as connections need to be adequately selected and maintained. Understanding fully how injury modulates the environment of the spinal cord and how this might affect the integration of new circuits will be crucial to furthering our progress.

When studying motor patterns as a measure of recovery, it may be helpful to tailor these to exercises which utilise the rubrospinal tract. As this is likely one of the main pathways for recovery of function, the focus of certain studies could be misleading [50]. Rats have been found to provide an accurate model for studying human SCI, with an analogous relationship with respect to functional, electrophysiological and morphological outcomes [51]. However, when improving upon the details of the therapy more studies on primates and humans would be useful. Another problem is that interactions between Nogo and its multiple receptors are not yet fully understood. Developing our understanding of them could lead to better treatments being developed, potentially giving greater alleviation of the growth inhibition. One group looked to the olfactory system which is one part of the CNS where extensive neuroplasticity is present under normal conditions. This study identified a key molecule in lateral olfactory tract formation which suppressed Nogo-NgR1 interaction [52]. Understanding the mechanism by which LOTUS inhibits Nogo could help develop the antibody treatment. Likewise, LOTUS itself or derivatives could be used complementarily with the antibody treatment. As NgR1 is a common receptor for most of the molecules in the pathway, it would make a reasonable target for antibody treatment. One group had attempted to assess this with an antibody highly selective for NgR1 [53]. Unfortunately, this produced only modest increases in functional recovery. Larger improvements were seen with both peptides targeting NgR anti-Nogo antibodies [54]. This could be due to cross-activation of the other receptors in the pathway and perhaps blocking a larger portion of them could achieve the effect the group aimed for. Nonetheless, the result was surprising and highlights the lack of understanding in this field.

In contrast to this, the identification of CSPGs as ligands for NgR1 and NgR3 proved more useful. A study found that the combined treatment using both anti-Nogo antibodies and chondroitinase was more effective than either on their own following a lesion [55]. The rats receiving combination therapy scored better on both the staircase and ladder walk test. Digesting CSPGs should increase regeneration itself as well as potentially enhancing the disinhibition of Nogo. Interestingly the antibodies stimulated the growth of more large axons whereas chondroitinase increase the number of fine fibres. This points to their difference in function in limiting CNS growth as well as diminishing inhibitory signals.

Finally, as recovery is usually dependant on the amount of spared tissue, large lesions tend to have worst outcomes. This could be improved by pairing antibody treatment with bridging strategies such as cell grafts. Furthermore, overcoming the inhibition of the glial scar or supplying the spinal cord with growth factors could have additive effects.

## Cortical side effects

Finally, and perhaps most importantly, the inhibition imposed on the CNS is there for a reason. As was discussed earlier, the likely function of CNS inhibition is stabilisation of adult connections. Another potential function was deduced from mice expressing elevated levels of NgR1 which had impaired long-term memory [56]. Administration of soluble Nogo reproduced this and suppressed long term potentiation (LTP) in the hippocampus [57]. Could this mean that Nogo treatment could affect behaviour, and destabilising connections could potentially lead to aberrant pathways and behaviour such as seen in Wahl et al. [42]. None of the other studies using anti-Nogo-A reported any side effects, but there is evidence to suggest that negative long-term effects of upregulated plasticity exist. Hyperactivation of the mTOR pathway by deletion of the regulator PTEN leads to increased regenerative sprouting in the CNS. When this was applied following optic nerve injury a large number of fibres were misguided and followed completely incorrect paths [58]. When the same method was repeated in granule cells of the dentate gyrus, aberrant connections formed similar to those in epilepsy and as expected the rats developed spontaneous seizures after 4 weeks [59]. More recently in the study carried out by Akbik et al. it was noted that the increased dendritic turnover in the cortex in Nogo knockout mice led to greater fear extinction on the second day of hearing unpaired tones [21]. Furthermore, variants of Nogo and NgR1 have been associated with the development of schizophrenia in animal models as well as genetic comparisons of human variants of the gene [60].

These could be potentially devastating side effects for patients treated with anti-Nogo mAbs. If such side effects are observed one way to overcome them would be to confine the antibody treatment to the spine and brain stem. As we have seen from Wyss et al. increased cortical reorganisation is not likely to be responsible for the improvement in recovery [33]. Therefore if the antibodies could be confined to the brainstem and spinal cord the therapy should be just as effective. However, if the antibodies do not reach the cortex behavioural changes or schizophrenia are very unlikely to be seen. Using complementary therapies such as cell grafts or glial scar inhibition would be ideal as they are confined to the spinal cord. This could mean lower amounts of antibodies could be used to obtain the same functional recovery. This could limit the side effects caused by lack of Nogo-mediated inhibition in the cortex. Perhaps differences will be identified in the pathway between the brain and spinal cord and can be utilised to tailor the treatment. Already established differences between the cord and cortex could also be exploited for drug activation or altering its activity.

## Final remarks

The Nogo pathway is most likely responsible for the stabilisation of CNS circuitry during adolescence. As such it downregulates CNS plasticity and thus the potential for functional recovery following damage to the spinal cord.

The use of anti-Nogo targeting antibodies has been well established to improve anatomical and functional recovery. Most of the acutely treated rats and monkeys show major recovery in function with some manual dexterity tests revealing no significant difference to healthy rats. The treatment has also shown to improve associated deficits in function such as spasms and bladder dysfunction with no reported side effects such as allodynia or hypersensitivity.

It is now believed that the major way by which suppressing Nogo increases functional recovery is the increased sprouting and bypass pathways in supraspinal and intraspinal circuitry. Most prominently the increase in fibres crossing over at the level of the red nucleus as well as the integration of propriospinal neurones into motor pathways. Though axonal regeneration through the site of the lesion has been consistently demonstrated; this has not been deemed to contribute significantly to the outcome.

Current evidence suggests most noticeable results after acute therapy with smaller yet significant effects on recovery with the treatment of chronic SCI. This recovery was most effective when followed with suitable dexterity training. Increased plasticity in itself is not useful unless it can be restrained and the new connections effectively utilised. Furthermore, competition for space during cortical remapping has been shown to result in permanent deficits in untrained tasks. Therefore, training should focus on a broad range of motor patterns. Altogether more investigation will be needed to establish the ideal regime for treatment and training.

Current development of the treatment would also benefit from a further study of the interactions of the Nogo pathway as well as the precise mechanisms by which recovery is achieved. Moreover, it is becoming clear that the role of Nogo is limited because of other environmental inhibitors associated with the scar. The extracellular matrix consists of CSPGs and tenascins, organized into a lattice-like structure called perineuronal nets. After SCI, progressive neurodegeneration occurs through inflammation of these nets, neuronal and glial apoptosis, glutamate excitotoxicity, potential disruption of the blood brain barrier, axonal demyelination and reactive astrogliosis, which all lead to the formation of an inhibitory glial scar [61]. This glial scar contributes to the inhibitory environment existing after injury independent of Nogo. For example, purified, recombinant myelin-associated glycoprotein (MAG) and oligodendrocyte myelin glycoprotein have been shown to collapse axonal growth cones, induce growth cone retraction and inhibit neurite outgrowth [62, 63]. Furthermore, the inflammatory NF-κB pathway is upregulated downstream of tumor necrosis factor-α, by glial cells and infiltrated immune cells, producing pro-inflammatory cytokines, chemokines and activates astrocytes to form the glial scar which hampers axonal growth across the lesion [64]. As such, by understanding the environmental changes that inhibit plasticity and repair following SCI, we can identify further targets for therapeutic intervention.

Lastly, Nogo has repeatedly been demonstrated to affect LTP and memory formation in the hippocampus with suppression of the pathway leading to increased fear extinction. Moreover, the Nogo pathway has been linked to the development of schizophrenia and increased sprouting in the dentate gyrus has been associated with epilepsy. Therefore, behavioural changes should be studied to prevent these potential side effects from developing.

## Conclusions

In conclusion, treatment of SCI with anti-Nogo antibodies looks to be moderately promising. It has consistently demonstrated to improve functional recovery after acute application with no reported side effects, although genetic evidence for its efficacy is mixed. Although plasticity increases greatly, we need to better our understanding of how the new connections are integrated in order to improve overall outcome. The therapy is also unlikely to be as effective with more extensive damage as spare tissue, and therefore potential bypass neurones, are limited. Lastly, there is growing evidence suggesting inhibition of the Nogo pathway could have seriously debilitating effects in the cortex. It is highly unlikely that a single target immunotherapy (e.g. anti-Nogo) will be successful; novel therapeutic strategies must confront issues with the extracellular environment of the CNS to promote both axonal regeneration and functional recovery. With the treatment soon re-entering clinical trials [65] it will be easier to assess these issues, as effects of the treatment are studied more thoroughly in humans. Provided these problems can be solved, overcoming the permanently debilitating effects of SCI could be a possibility in the very near future.

## Data Availability

NA

## Abbreviations

CNS: Central nervous system
CSPG: Chondroitin sulphate proteoglycans
LTP: Long term potentiation
Nogo: Nogo-A, Nogo-B, Nogo-C
ROCK: Rho kinase
SCI: Spinal cord injuries
